# Supplementary material for: Content-rich biological network constructed by mining PubMed abstracts
Source: BMC Bioinformatics. 2004 Oct 8;5:147. doi: 10.1186/1471-2105-5-147 (PMC528731; doi:10.1186/1471-2105-5-147)
Supplement: Additional File 2 — The original results of the above study (non-essential files are deleted to keep the file size under the limit set by BMC bioinformatics). [file 1471-2105-5-147-S2.bz2 › chilibotAdditionalFile2/dip05/283ID7744247E1106/html/CDC25B_RAF1.html]

 


 **CDC25B** and **RAF1** 
  
Found 2 abstracts in PubMed, retrieved 2.  
 

 What does Google say? 
 PDF only 
| .edu only 

---

**Interactive relationship** (e.g. stimulation, inhibition, etc)

**Non-interactive relationship** (e.g. studied together, co-existance, homology, etc.)

- Thus, we investigated whether the  **Raf1** , Mdm2, c Myc, Cdc25a and  **Cdc25b**  proto oncogenes, which are implicated in the ras or p53 or pRb pathways, are alternative oncogenic target genes.  Ref: 10894886 Mutat Res, 2000
- Genetic analysis of  **Raf1** , Mdm2, c Myc, Cdc25a and  **Cdc25b**  proto oncogenes in dideoxycytidine and butadiene induced lymphomas in B6C3F1 mice.  Ref: 10894886 Mutat Res, 2000
